# Herpes Simplex Virus 1 Induces Brain Inflammation and Multifocal Demyelination in the Cotton Rat Sigmodon hispidus

**DOI:** 10.1128/JVI.01161-19

**Published:** 2019-12-12

**Authors:** Marina S. Boukhvalova, Emma Mortensen, Aissatou Mbaye, Diego Lopez, Lorne Kastrukoff, Jorge C. G. Blanco

**Affiliations:** a Sigmovir Biosystems, Inc., Rockville, Maryland, USA; b The University of British Columbia, Department of Medicine, Vancouver, BC, Canada; University of Southern California

**Keywords:** HSV-1, demyelination

## Abstract

Demyelinating central nervous system (CNS) disorders like multiple sclerosis (MS) and acute disseminated encephalomyelitis (ADEM) have been difficult to study and treat due to the lack of understanding of their etiology. Numerous cases point to the link between herpes simplex virus (HSV) infection and multifocal CNS demyelination in humans; however, convincing evidence from animal models has been missing. In this work, we found that HSV-1 infection of the cotton rat Sigmodon hispidus via a common route (lip abrasion) can cause multifocal CNS demyelination and inflammation. Remyelination occurred shortly after demyelination in HSV-1-infected cotton rats but could be incomplete, resulting in “scars,” further supporting an association between HSV-1 infection and multifocal demyelinating disorders. Virus was detected sequentially in the lip, trigeminal ganglia, and brain of infected animals. Brain pathology developed primarily on the ipsilateral side of the brain stem, in the cerebellum, and contralateral side of the forebrain/midbrain, suggesting that the changes may ascend along the trigeminal lemniscus pathway. Neurologic defects occasionally detected in infected animals (e.g., defective whisker touch and blink responses and compromised balance) could be representative of the brain stem/cerebellum dysfunction. Immunization of cotton rats with a split HSV-1 vaccine protected animals against viral replication and brain pathology, suggesting that vaccination against HSV-1 may protect against demyelinating disorders.

**IMPORTANCE** Our work demonstrates for the first time a direct association between infection with herpes simplex virus 1, a ubiquitous human pathogen generally associated with facial cold sores, and multifocal brain demyelination in an otherwise normal host, the cotton rat Sigmodon hispidus. For a long time, demyelinating diseases were considered to be autoimmune in nature and were studied by indirect methods, such as immunizing animals with myelin components or feeding them toxic substances that induce demyelination. Treatment against demyelinating diseases has been elusive, partially because of their unknown etiology. This work provides the first experimental evidence for the role of HSV-1 as the etiologic agent of multifocal brain demyelination in a normal host and suggests that vaccination against HSV-1 can help to combat demyelinating disorders.

## Abstract (precis)

Our work demonstrates for the first time a direct association between infection with herpes simplex virus 1, a ubiquitous human pathogen generally associated with facial cold sores, and multifocal brain demyelination in an otherwise normal host, the cotton rat Sigmodon hispidus. For a long time, demyelinating diseases were considered to be autoimmune in nature and were studied by indirect methods, such as immunizing animals with myelin components or feeding them toxic substances that induce demyelination. Treatment against demyelinating diseases has been elusive, partially because of their unknown etiology. This work provides the first experimental evidence for the role of HSV-1 as the etiologic agent of multifocal brain demyelination in a normal host and suggests that vaccination against HSV-1 can help to combat demyelinating disorders.

## INTRODUCTION

Herpes simplex virus 1 (HSV-1) is a ubiquitous pathogen most commonly associated with cold sores around the mouth and ocular infections. Primary HSV-1 infection is usually acquired during the first 2 decades of life. An estimated 67% of people under age 50 have been infected with HSV-1 worldwide (1). The virus is a highly successful pathogen that normally does not impair the health of the individual and establishes latency in the host for a duration of a lifetime. HSV-1 must come into contact with abraded skin or mucosa for infection to occur (2). Upon initial infection, the virus replicates in the epidermis or epithelial mucosa, gaining access to the sensory endings of the nerve innervating the primary site of infection. From there, the virus can travel in a retrograde mode to reach sensory ganglia, where it establishes latency. HSV-1 can reactivate from latency in response to a variety of stimuli, including stress, exposure to UV light, immunosuppression, termination of antiviral treatment, or hormonal fluctuations. During reactivation, the virus travels along the axon in anterograde mode, replicating in the tissue of the dermatome innervated by the sensory neuron in which the virus established latency. Ulcerative lesions or asymptomatic shedding at peripheral sites may lead to further transmission of virus.

Although HSV-1 normally induces a mild primary infection, it can be associated with serious sequelae, such as herpes simplex encephalitis (HSE), meningitis, and meningoencephalitis (3). The mortality associated with untreated HSE is about 70%, with 97% of survivors unable to return to their normal functioning level (4–7). Current antivirals and supportive therapy reduce HSE-associated 1-year mortality to 5% to 15% (8–10). The neuropsychiatric deficits, however, remain a problem for those who recover. Computed tomography (CT) reveals abnormal areas in the brain of 25% to 80% of patients with HSE if they are imaged shortly after admission (11, 12). The findings normally include hypodense regions, edema, or areas of increased contrast (13–15). Perivascular lymphocytic cuffing in pons, midbrain, and temporal lobe, along with multiple petechial hemorrhages and ecchymosis, has been noted in association with HSE (16). Thalamic, brain stem, and cerebellar involvement in HSE has also been described (17).

Several types of animals have been used to model HSV-1 infection and HSE (18, 19). Mice, rabbits, and guinea pigs are used the most to study immune mechanisms of HSV-1 infection and the process of HSV latency and reactivation (20–23). For example, studies in a murine model suggest that HSV-1 can reside in the brain of latently infected animals and can get reactivated locally (22, 24–26). The extent of direct reactivation of HSV-1 in the central nervous system (CNS) versus transport of reactivated virus from peripheral ganglia, however, continues to be a subject of discussion (23). Recurrent HSV-1 infection in mice was shown to contribute to neurodegeneration and cognitive deficits, providing additional evidence for a growing association between latent HSV infection and Alzheimer’s disease (AD) (27, 28). In the experimental challenge models of acute HSV infection, virus has been inoculated by different routes, including intraperitoneal, oral or nasal mucosa, flank skin, rear footpads, or direct injection into brain. Injection of HSV-1 into the brain of mice was shown to lead to encephalitis, with inoculation of HSV into the hippocampus area yielding more severe disease than inoculation into the cerebellum (29). Inoculation of HSV-1 into the olfactory bulb of rabbits induced localized infection, with involvement of the temporal cortex and frontal lobes (30). Injection of virus into the whisker area of dark agouti (DA) rats caused encephalitis in the brain stem and olfactory bulb (31). Cases of natural HSV-1 infection have been reported in rabbits, with signs of encephalitis and neuronal and glial necrosis in the cerebral cortex (32, 33).

In addition to HSE and AD, HSV-1 infection may be associated with debilitating CNS disorders characterized by multifocal demyelination, including multiple sclerosis (MS) and acute disseminated encephalomyelitis (ADEM), but the connection has been disputed. MS and ADEM belong to the group of disorders called idiopathic inflammatory and demyelinating diseases (IIDD), with the word “idiopathic” emphasizing their unknown cause. Multiple studies provide evidence that in humans HSV infection/encephalitis and MS or ADEM are connected (34–38); however, evidence from animal models of HSV infection is lacking to support that association. MS is associated with the appearance of myelin lesions in the CNS. Characteristics of MS lesions include demyelination, inflammatory cell infiltration, and relative preservation of axons; however, in some patients, axonal degeneration and neuronal loss are prominent features. The remyelination occurs during early phases of the disease, although it is usually incomplete (39, 40). Ongoing de- and remyelination are often detected during early MS (41, 42).

The actual name of MS refers to the scars forming in the nervous system, primarily the white and gray matter of the brain stem, optic nerve, basal ganglia, corpus callosum, thalamus, hippocampus, and spinal cord. MS often has to be distinguished from ADEM, with the symptoms and appearance of brain imaging during ADEM and the first attack of MS showing similarities. However, ADEM is typically characterized by a single attack of expansive myelin damage, while MS may involve numerous attacks over time. Generally, detection of older brain lesions on magnetic resonance imaging (MRI) suggests that the condition may be MS rather than ADEM. ADEM can progress to a relapsing remitting form, and the distinction between recurrent ADEM and MS is a subject of debate (43).

An animal model of HSV-1-induced multifocal CNS demyelination has been attempted in the search for a suspected link between HSV-1 infection and MS. In 1979, it had been reported that Swiss mice infected with HSV-1 after scraping the skin of a nose with a fine needle develop lesions in the areas of the brain stem (BST) corresponding to the trigeminal root entry zone (TREZ) and trigeminal tracts and nuclei (44). Disruption of myelin sheaths, inflammatory cell infiltration, and macrophages loaded with myelin degradation products were present in the lesions. Lesions were limited to BST, however, and no multifocal demyelination was found (44). CNS infection after lip inoculation of HSV-1 was shown to be highly dependent on the choice of murine strains. C57BL/6J mice were found to be the most resistant in regard to progression to CNS, followed by BALB/c/cByJ and A/J mice (45). HSV-1 infection in C57BL/6J was limited to the pons, with inflammatory cells detected at the TREZ area and no lesions. In moderately resistant BALB/cByJ mice, unifocal demyelinating lesions in BST were seen. In A/J animals, however, multifocal demyelinating lesions throughout the brain were detected (45). In addition to A/J mice, CNS demyelinating lesions also developed in SJL/J and PL/J mice inoculated via the oral mucosa (46). The connection between HSV-1 infection and major demyelinating disorders in humans still remained elusive, however. The susceptible murine strains A/J, SJL/J, and PL/J, in which multifocal demyelination was detected, have numerous defects in complement system, macrophage function, and/or muscle repair and are used to study developmental defects, epilepsy, spontaneous tumorigenesis, myopathy, and/or autoimmunity. These defects may affect the pathogenesis of HSV-1 infection and its CNS manifestation.

In this work, we used cotton rats, Sigmodon hispidus (47–49), to model lip HSV-1 infection and found that HSV-1 infection can cause inflammation and multifocal demyelination in the CNS. Remyelination occurred shortly after demyelination in HSV-1-infected cotton rats but could be incomplete, resulting in “scars,” supporting an association between HSV-1 infection and MS and/or ADEM. This work may present the first experimental evidence of multifocal demyelinating disease induced by lip HSV-1 infection in an otherwise normal animal and provide much-needed experimental evidence for infectious etiology of IIDD. Importantly, this work demonstrates that CNS demyelination caused by HSV-1 infection can be prevented by vaccination against HSV-1.

## RESULTS

### Dynamics of HSV-1 infection in cotton rats, S. hispidus, after lip abrasion.

To evaluate HSV-1 infection in cotton rats, animals were inoculated with HSV-1 strain 17 via abrasion in the vermilion border of the right side of the upper lip and monitored for 1 month after infection. HSV-1 infection was associated with moderate mortality/morbidity, primarily during the second and third weeks postinfection (Table 1). Mortality/morbidity was, in general, higher for younger animals than for the older ones (13% versus 6% average mortality/morbidity for females age 5 to 6 and 10 to 12 weeks, respectively; 20% versus 12% average mortality/morbidity for males age 5 to 6 and 10 to 12 weeks, respectively) and appeared to be more pronounced in male animals (the differences were not statistically significant, though). Hunched posture, compromised balance, or splayed feet were often detected on the same day or a few days before death, although some mortalities occurred without detectable morbidity. Milder symptoms in the form of defective blink and whisker touch responses and facial paralysis/asymmetry were seen in some of the infected animals (Table 2). Defects affected the right side of the face and/or the right eye, started at the end of the first week, and were resolved by the third week after infection. Head tilt to the right was among the mild symptoms from which animals could recover (Table 2) but was also occasionally seen in animals that succumbed to infection (Table 1). Occasionally, damage to the right lip by what appeared as excessive scratching was seen (Table 2). No blisters on the lip were detected in any of the animals. Young (5 to 6 weeks old) female animals were chosen for the majority of subsequent studies on dynamics and pathology of HSV-1 infection.

**TABLE 1:** Morbidity and mortality in cotton rats infected with HSV-1^*a*^

Study	Sex	Age (wks)	Virus dose (PFU)	Total no. of animals	Mortality		Morbidity		Demyelinated lesions^*c*^
					Day pi^*b*^ (no. of animals)	Mortality (%)	Day pi	Symptom(s)^*c*^	
1	Fem^*d*^	5	6 × 10^4^	65	9 (1)	8			NA
					13 (1)				
					16 (3)				
2	Fem	5	1 × 10^4^	12	10 (1)*	25	10	CB, SF	NA
					11 (1)*		10	CB, SF, HP	
					12 (1)*		11	CB, SF, HP, HT	
3	Fem	6	1.4 × 10^5^	19	9 (1)*	5	9	Paralysis, right side	BST, Cer, FBr
4	Fem	6–8	1.4 × 10^5^	40	9 (2)	15			NA
					13 (1)				
					20 (1)				
					21 (1)				
					25 (1)				
5	Fem	8	1.4 × 10^5^	8		0			NA
6	Fem	10	1.4 × 10^5^	28	3 (1)*	11		HP	NA
					10 (1)*		10	HP, CB	NA
					14 (1)*		14	CB, R-mvt, HT	NA
7	Fem	10	2.8 × 10^5^	10		0			NA
8	Fem	12	2.8 × 10^5^	12	7 (1)*	8	7	CB, HT	NA
9	Male	5	1.4 × 10^5^	28	9 (1)*	7	9	CB, jumpiness	NA
					10 (1)		7	SF	BST, Cer
10	Male	5	1.4 × 10^5^	12	10 (1)	33			NA
					10 (1)*		10	CB, SF	
					11 (1)*		10	CB, SF, HT	
					12 (1)*		11	CB, SF	
11	Male	10	1.4 × 10^5^	21	10 (1)	19			NA
					12 (1)*		10	HP, CB	BST, Cer, FBr
					17 (1)		14	HP, CB	BST
					17 (1)		17	CB	NA
12	Male	10	2.8 × 10^5^	10	17 (1)	10	14	HP, CB	NA
13	Male	12	2.8 × 10^5^	12	12 (1)*	8	12	CB, SF, HP, HT	NA

a Cotton rats between 5 and 12 weeks of age were infected with HSV-1 in the lip via abrasion and kept for 1 month after infection for general observation. Mortality was documented and reported here as the day postinfection on which it was detected and number of animals that died that day. Brain samples were collected for histopathology analysis from select animals who had to be sacrificed or who died recently, with the presence of demyelinated lesions indicated by the section of the brain in which they were detected. A summary of multiple studies run with female and male animals is shown.

b pi, postinfection; *, some animals exhibited morbidity and had to be sacrificed, with the nature of morbidity and the day it was detected described under the corresponding section of the table.

c BST, brain stem and pons; Cer, cerebellum; FBr, forebrain/midbrain; NA, histopathology not available; HP, hunched posture; CB, compromised balance; SF, splayed feet; HT, head tilt to the right side; R-mvt, right-side-biased movement.

d Fem, female.

**TABLE 2:** Mild symptoms of HSV-1 infection in cotton rats inoculated with HSV-1 via abrasion^*a*^

Sex	Animal no.	Symptom(s) according to day pi												
		0	3	4	5	6	7	10	11	12	13	14	17	19
Female	1					HT	HT	HT	HT	HT	HT	HT		
	2													
	3													
	4				LD									
	5					HT	HT, RT−	HT, FA, RT−	HT, FA, RT−					
	6						FA, RT−	RT−	RT−					
	7					HT	HT, FA, RT−	HT, FA, RT−	HT, FA, RT−	FA, RT−	FA, RT−	FA, RT−		
	8													
	9													
	10													
	11					FA	FA, RT−, RB−, HT	FA, RT−, RB−, HT	FA, RT−, RB−, HT	RT−	FA, RT−	RT−		
	12			LD										
	13													
	14													
	15													
													
Male	1					FA, HT	FA, RT−, HT, SF							
	2					FA, HT	FA, RT−, RB−, HT	FA, RT−, RB−, HT	FA, RT−, RB−, HT	FA, RT−, RB−	FA, RT−, RB−	FA, RT−		
	3							FA, RT−	FA, RT−	FA, RT−	FA, RT−	FA, RT−		
	4					HT	HT	HT	HT	HT	HT			
	5							FA	FA					
	6													
	7					HT	HT	HT	HT, RT−					
	8													
	9													
	10				LD		HT	HT	HT					
	11													

a Female and male cotton rats, 5 to 6 weeks old, were infected with 5 log_10_ PFU HSV-1 in the lip via abrasion and monitored for 19 days after infection for mild symptoms of infection. Symptoms included facial asymmetry (FA) with drooping right side of the face and nose turned to the left, defective right whisker touch response (RT−), defective right eye blink response (RB−), or head tilt (HT) to the right. Onset of mild symptoms was seen at the end of the first, beginning of second week after infection (day 6–10), with recovery from symptoms on day 17 or earlier. Some animals had right lip damaged (LD) by what appeared like excessive scratching. Gray cells indicate mortality. Male animal number 1 had splayed back feet (SF) 3 days before it was found dead. Experiment was repeated once, with similar results. Results of a representative experiment are shown.

**FIG 1:**
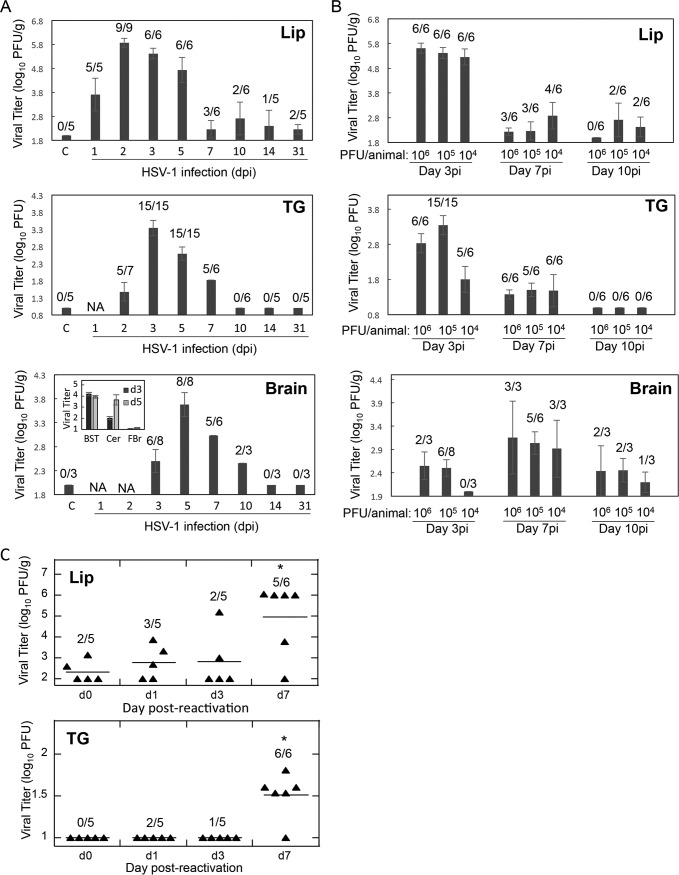
Viral detection during primary lip HSV-1 infection and after HSV-1 reactivation in cotton rats *S. hispidus*. (A) Female cotton rats (5 to 6 weeks old) were inoculated with 5 log_10_ PFU HSV-1 in the vermilion border of the upper lip on the right side via abrasion. Lip (right side of the upper lip), right trigeminal ganglion (TG), and brain (whole brain minus olfactory bulbs) were collected at various times after infection for analysis of viral titers by HSV-1 plaque assay. The experiment was run with 3 to 5 animals per time point and repeated at least once for select time points. Cumulative data for all animals analyzed at a particular time point are shown (geometric mean [GM] ± standard error [SE]). Ratios correspond to the number of animals with detectable virus to the total number of animals analyzed at that time point. C, control, uninfected animals; NA, not analyzed. Insert at the bottom shows viral titers (log_10_ PFU/g) detected in parts of brain (BST, brain stem; Cer, cerebellum; FBr, forebrain/midbrain) collected individually for viral titration on days 3 and 5 postinfection (4 animals per time point, GM ± SE). (B) Dose dependency of HSV-1 lip infection in cotton rats. Female cotton rats (5 to 6 weeks old) were inoculated with 4, 5, or 6 log_10_ PFU HSV-1 as described for panel A and sacrificed on days 3, 7, and 10 postinfection (dpi) for analysis of viral load in the lip, TG, and brain. Experiment was run with 3 to 5 animals per group and repeated for select doses/time points with data presented as described in the legend to panel A. (C) One month after original infection, animals infected as described for panel A were reactivated by injection of corticosteroid triamcinolone acetonide. Samples were collected for viral titrations at various times after reactivation. Experiment was run using 5 to 6 animals per time point. d0 corresponds to the time immediately before triamcinolone administration (1 month after the initial HSV-1 infection). Each symbol represents one animal, and the line across each group corresponds to GM value. Statistical significance of increase in viral titers after triamcinolone reactivation was assessed by Student’s *t* test. *, *P* < 0.05.

HSV-1 robustly replicated in the lip of cotton rats, with ∼5 to 6 log_10_ PFU per gram of tissue (PFU/g) detected on days 2 to 3 postinfection, followed by a slight reduction on day 5, and a decline to ∼2 log_10_ PFU/g on day 7 (Fig. 1A). Virus remained detectable in a few animals in each group at 10 days, 2 weeks, and 1 month after infection, but the level was low (less than 3 log_10_ PFU/g). There appeared to be a slight increase in HSV-1 lip titer between days 7 and 10 postinfection; however, the change was small and could have reflected residual replication after initial infection rather than spontaneous reactivation. HSV-1 appeared in the right trigeminal ganglion (TG) with a delay with respect to the timing of the HSV-1 increase in the lip. Virus was barely detectable in TG on day 2 postinfection, when the amount of HSV-1 in the lip was already high. HSV-1 titer in TG, however, rose sharply thereafter, reached ∼3 log_10_ PFU on day 3 postinfection, and remained elevated until day 5, before diminishing on day 7 and returning to undetectable on day 10 postinfection. In contrast to the lip, no virus was detected in TG at 2 weeks or 1 month after HSV-1 infection. Dynamics of HSV-1 appearance in the brain was further offset with respect to increases in the lip and TG HSV-1 loads. Virus became detectable in the brain on day 3 postinfection, increased in titer on day 5 postinfection, and declined gradually on days 7 and 10 postinfection, becoming undetectable 2 weeks after infection (Fig. 1A). To determine the dynamics of virus appearance in different parts of the brain, brain stem, cerebellum, and forebrain/midbrain were collected individually from HSV-1-infected animals on days 3 and 5 postinfection and viral load was quantified by plaque assay (Fig. 1A, insert in the “Brain” panel). A large amount of virus was present in the brain stem on both days of analysis, with 3.77 ± 0.16 log_10_ PFU/g and 3.52 ± 0.16 log_10_ PFU/g detected on days 3 and 5, respectively (*n* = 4 per time point, 4/4 animals with detectable virus in the brain stem on both days). The amount of virus in the cerebellum increased between days 3 and 5 from 1.61 ± 0.16 log_10_ PFU/g on day 3 (*n* = 4, virus detectable in 4/4 animals) to 3.25 ± 0.46 log_10_ PFU/g on day 5 (*n* = 4, virus detectable in 4/4 animals). A very small amount of virus (single plaques in undiluted samples) was also detected in the forebrain/midbrain of 50% of animals (2/4) analyzed on day 3 and 100% of animals analyzed (4/4) on day 5. These data suggest a gradual progression of HSV-1 from brain stem to cerebellum, with the appearance of a small quantity of detectable virus in the forebrain/midbrain on days 3 to 5 postinfection. The dose dependency of HSV-1 load in the lip, TG, and brain was evaluated next (Fig. 1B). Animals were infected with 6, 5, or 4 log_10_ PFU of virus. The volume of virus inoculum was kept constant at 20 μl for each dose inoculum. A moderate dose-dependent reduction in viral titer was detectable on day 3 postinfection in the lip, TG, and brain.

To investigate whether HSV-1 can be reactivated in cotton rats, HSV-1-infected animals were allowed to convalesce for 1 month after the original infection and were treated with high-dose glucocorticoid (triamcinolone). Triamcinolone treatment caused a significant increase in HSV-1 titer in the lip and TG on day 7 postreactivation (Fig. 1C). Studies with triamcinolone-treated animals could not be extended beyond day 7 postreactivation because of the detrimental effect of the high-dose glucocorticoid (40 mg/kg) associated with significant morbidity/mortality. This large dose of corticosteroids was required for the uniform reactivation of virus in the model. Preliminary studies conducted using a lower dose of triamcinolone demonstrated that reactivation can also be achieved with this dose of steroid, although it was of a much more sporadic nature, with only a fraction of animals showing reactivation. Further studies are needed to optimize conditions of HSV-1 reactivation in the model and to determine whether the brain may be the site of HSV-1 reactivation, as has been suggested by the murine model (22–26). In spite of these limitations, results of reactivation studies presented here show that HSV-1 establishes latency and can be reactivated in HSV-1-infected cotton rats.

### Brain histopathology in HSV-1-infected cotton rats.

Because primary infection with HSV-1 appeared to be associated with some neurologic manifestations and because virus was detected in the brain, samples of the brain were collected for histopathology analysis. Brains were extracted, fixed in 10% buffered formalin, and cut into four coronal sections. The majority of changes were detected in the sections corresponding to (i) hindbrain at the level of the facial nucleus or the inferior olive (called cerebellum and BST/pons here) and (ii) forebrain at the level of the caudal end of the thalamus and midbrain (called forebrain/midbrain here).

**FIG 2:**
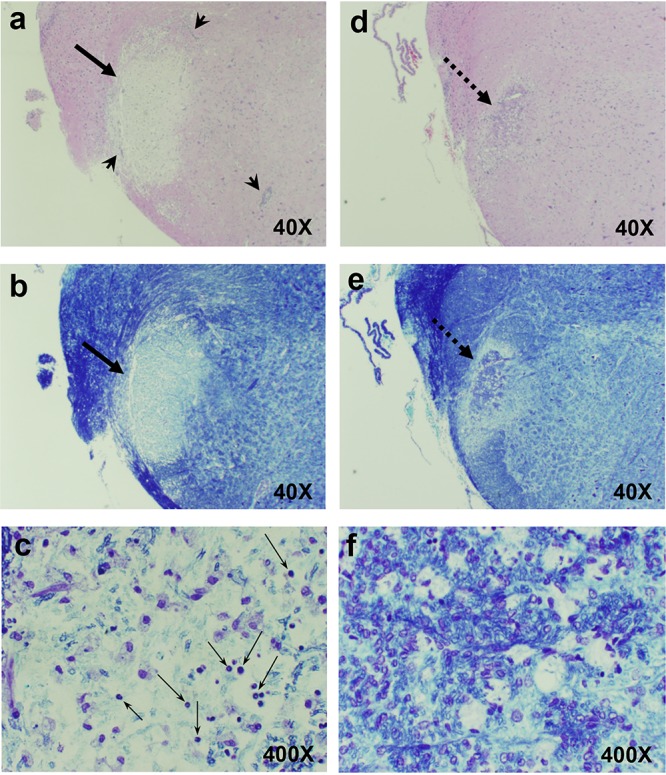
Demyelinated brain lesions visualized with hematoxylin and eosin (H&E) or Luxol fast blue (LFB) stains. Examples of two types of BST/pons lesions in cotton rats infected with HSV-1 as described in the legend to Fig. 1A are shown, including an acute lesion 1 week postinfection (a, b, and c) and an older lesion 1 month postinfection (d, e, and f) stained with H&E (a and d) or LFB (b, c, e, and f). Older lesions (d and e, thick dashed arrows) appeared to have denser centers than acute lesions (a and b, thick solid arrows). Arrowheads in panel a point to perivascular cuffing. Higher-magnification view of the center of an acute lesion (c) shows dramatic loss of visible myelin and presence of infiltrating granulocytes and monocytes (thin arrows). Older lesions lacked visible infiltrating cells and had deposits of LFB-positive material that formed net-like structures (f). Magnification of each image is shown in the bottom right corner.

Among the first detectable changes in the brain of HSV-1-infected cotton rats was a significant disruption of myelin fibers (demyelinated lesions) in the BST/pons, often accompanied by infiltration of inflammatory cells around blood vessels (perivascular cuffing) (Fig. 2). The majority of BST/pons lesions were located in the right part of the BST/pons, the side ipsilateral to infection. The areas corresponding to (at different level of cuts) the sensory root of the trigeminal nerve, TREZ, and principal and spinal trigeminal nuclei were affected. Additional lesions could be seen in what may correspond to solitary tract and spinal trigeminal tract, as well as in areas corresponding to ipsilateral dorsal trigeminothalamic or mesencephalic trigeminal tracts. Demyelinated lesions were pale and could be visualized with either regular hematoxylin and eosin (H&E) stain or myelin-specific Luxol fast blue (LFB) stain (Fig. 2).

**FIG 3:**
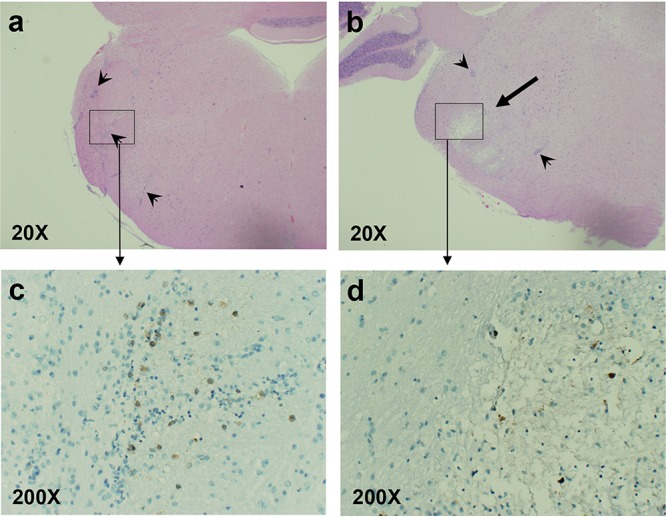
Detection of HSV-1 antigens in the brain lesions of cotton rats infected with HSV-1. Cotton rats were infected with HSV-1 as described in the legend to Fig. 1A and sacrificed on day 5 (a and c) or day 7 (b and d) postinfection. Brain sections corresponding to the right side of BST/pons stained with H&E (a and b) or anti-HSV-1 antibody (c and d) are shown. (a) Arrowheads point to perivascular cuffing. No lesions were yet visible on day 5 postinfection, although extensive pervasculitis was already detectable. (b) Demyelinated lesions became apparent on day 7 postinfection (thick arrow). (c and d) Immunohistochemistry (IHC) of BST/pons areas boxed in panels a and b, respectively, indicates the presence of HSV-1 antigens (brown color). HSV-1 antigens were dispersed throughout the area of inflammation on day 5 postinfection (c). On day 7 postinfection (pi) (d), HSV-1-infected cells were detected by IHC in the lesion but not in the adjacent intact area of the brain. Magnification of each view is shown in the bottom left corner. IHC was performed on brain sections from 4 different animals, with representative results shown.

The morphology of lesions during early and later stages of infection was different. Acute lesions detected on day 7 postinfection had a dramatic disruption of white matter and infiltration with mononuclear cells and granulocytes (Fig. 2a to c). These lesions contained small, shredded fragments of LFB-positive myelin among remnant-covered axons, neuronal cell bodies, and other cells widely separated by clear space (Fig. 2c). Lesions present 2 weeks to 1 month after infection had an appearance of partially remyelinated structure and lacked visible infiltrating cells (Fig. 2d to f). These “late” or “regenerating” lesions were paler than normal tissue but had more LFB-positive material than acute lesions. Regenerating lesions had disordered, thick, and thin deposits of LFB-positive material that formed crude, net-like structures, suggesting poor or incomplete myelin repair (Fig. 2f). Although further evidence of remyelination would require electron microscopy studies, late demyelinated lesions could be distinguished from acute lesions under relatively low magnification (40×) and remained visible throughout the time of analysis as “scars” or “shadow” plaques. Both acute and regenerating demyelinated brain lesions could sometimes be detected in the same HSV-1-infected animal during the second week after infection. At the time when pons/BST lesions were highly visible (day 7 postinfection), HSV-1 antigens were detectable within the lesion and at the edge of the lesion (Fig. 3). Intact tissue adjacent to the lesion did not show any HSV-1-specific staining. Two days before lesion formation (day 5 postinfection), HSV-1 antigens could be seen in large cells in pons/BST in the area where demyelinated lesions were to develop (Fig. 3). Immunohistochemistry was not performed on other parts of the brain.

**FIG 4:**
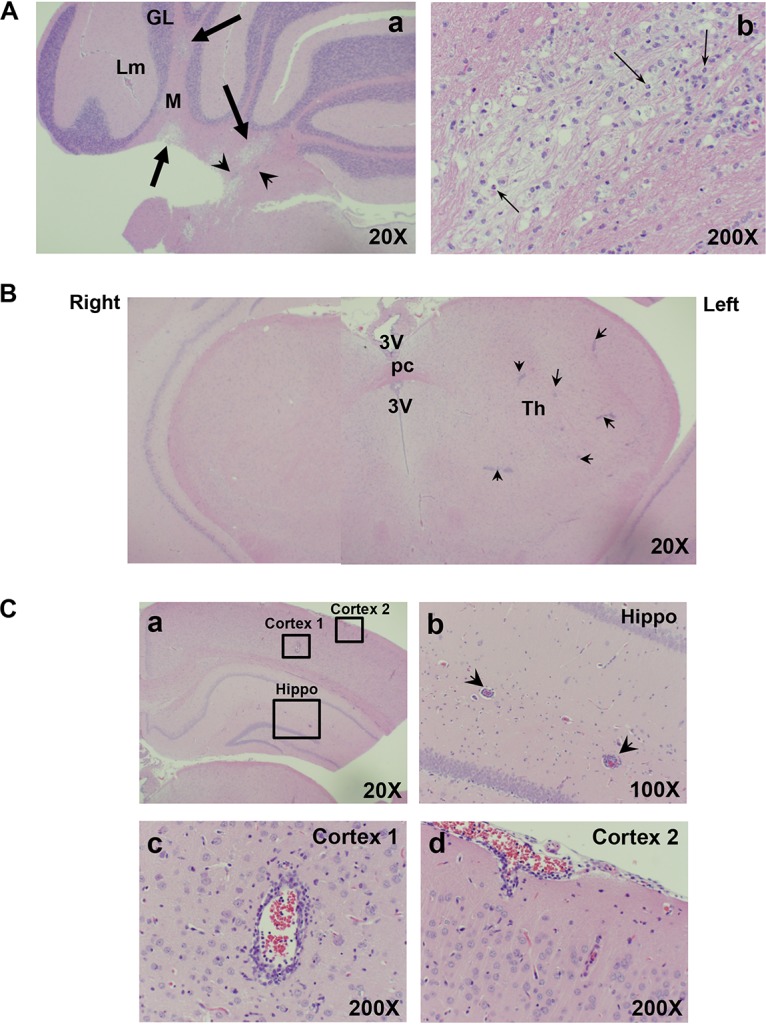
Changes in the cerebellum and forebrain/midbrain of HSV-1-infected cotton rats. Animals were infected with HSV-1 as described in the legend to Fig. 1A. (A) (a) Right side of the cerebellum of HSV-1-infected cotton rat sacrificed 7 days after infection. Commonly detected changes included demyelinated lesions (thick arrows), perivascular cuffing (arrowheads), and leptomeningitis (Lm). M, medulla; GL, granular layer. (b) Higher-magnification view of the demyelinated lesion shows infiltrating granulocytes (thin arrows). (B) Changes in the forebrain/midbrain of HSV-1-infected animal sacrificed 10 days after infection. Arrowheads indicate perivascular cuffing. Changes in the forebrain/midbrain tended to be located predominantly on the left side of the brain, the side contralateral to the side of infection. 3V, third ventricle; pc, posterior commissure; Th, thalamus. (C) (a) An example of rarely detected inflammation in the hippocampus and cortex of HSV-1-infected animal sacrificed on day 10 postinfection. (b, c, and d) Higher-magnification views of hippocampus (Hippo) and Cortex 1 and Cortex 2 areas boxed in panel a. No changes were detected in the right-side hippocampus and cortex of this animal (not shown).

Extensive demyelinated lesions were also visible in the cerebellum. These lesions were located mostly in the medullar region and were morphologically different from lesions in the BST/pons (Fig. 4A). Lesions with severely disrupted centers were scarce. There appeared to be fewer infiltrating cells than in early BST/pons demyelinated lesions. Repaired lesions were visible in the cerebellum at 2 weeks after infection. One of the most pronounced inflammatory changes in the cerebellum was inflammation in leptomeninges. It peaked 1 week postinfection and remained elevated throughout the course of infection.

In addition to changes in the cerebellum and pons of infected animals, changes were also occasionally detected in the forebrain/midbrain. These changes were more of an inflammatory nature than the disruption of tissue by demyelinating lesions. Perivascular cuffing in the forebrain/midbrain could be seen in some animals starting several days after infection and was predominant on the left side of the brain, contralateral to the side of infection (Fig. 4B). The left-side location of inflammation in the forebrain may be associated with the course of the trigeminal lemniscus pathway that crosses over to the other side of the brain, opposite to the side of the body on which sensory information is received. On at least one occasion, inflammation in the hippocampus and cortex (including leptomeninges) was also seen (Fig. 4C).

**FIG 5:**
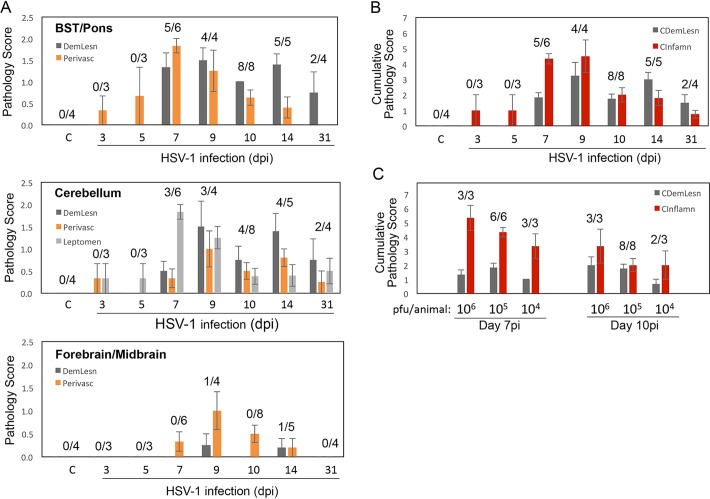
Time course and dose dependency of brain histopathology in HSV-1-infected cotton rats. (A and B) Brain histopathology scores in cotton rats infected with HSV-1 as described in the legend to Fig. 1A, time course of changes. On various days after infection, brains were collected and H&E slides prepared for histopathology analysis. (A) Demyelinated lesions (DemLesn), perivascular cuffing (Perivasc), and leptomeningitis (Leptomen, cerebellum only) were evaluated in BST/pons, cerebellum, and forebrain/midbrain and are presented for each brain area separately. (B) Cumulative histopathology scores across the three brain areas for the same type and number of brain samples as shown in panel A. Cumulative demyelinated lesions score (CDemLesn) includes DemLesn scores in BST/pons, cerebellum, and forebrain/midbrain. Cumulative inflammation score (CInflamn) includes leptomeningitis score in cerebellum and perivascular cuffing scores in cerebellum, BST/pons, and forebrain/midbrain. (C) Effect of virus challenge dose on cumulative brain histopathology in HSV-1-infected cotton rats. Female 5- to 6-week-old animals were inoculated with 4, 5, or 6 log_10_ PFU HSV-1 in the lip and sacrificed on days 7 and 10 for analysis of cumulative brain histopathology. Ratios correspond to the number of animals with demyelinating lesions detected in individual sections of the brain (A) or any section of the brain (B and C) to the total number of samples analyzed at that particular time point or dose of infection (C). Experiment was run with 3 to 4 animals allocated for brain histopathology analysis per time point and repeated at least once for select time points, with results from at least 2 independent experiments shown. Data are shown as mean ± SE for all animals in a group.

The dynamics of brain histopathology changes clearly indicated the appearance of inflammation before the development of demyelinated lesions (Fig. 5A and B) and a dependency of brain pathology on the dose of challenge virus (Fig. 5C). Demyelinated lesions were first detected on day 7 postinfection and continued to be visible throughout the study, albeit, as mentioned earlier, their format changed from acute lesions to partially “remyelinated” lesions over time. Some lesions might have undergone a more complete remyelination, as no such lesions could be seen in some of the infected animals 1 month after infection, while all animals displayed lesions during the second week after infection. Inflammation started a few days after infection and was maximal on days 7 to 9 postinfection. The appearance of lesions after corticosteroid reactivation did not change significantly from what was seen 1 month after infection (data not shown). There were no new lesions with inflammatory cell infiltrates. However, these animals were treated with a high dose of corticosteroids, and it is not clear how the anti-inflammatory effect of corticosteroid treatment could have affected lesion dynamics. No additional studies were conducted to investigate lesions in triamcinolone-treated animals, as conditions for reactivation need to be further optimized.

**FIG 6:**
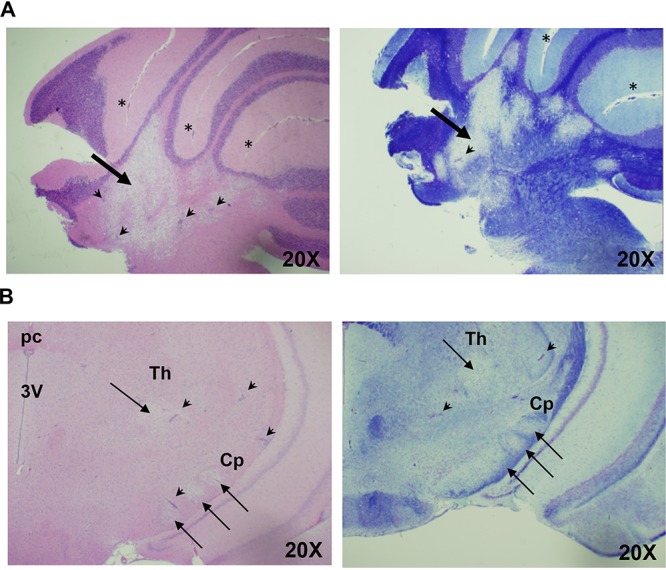
Histological changes in the brain of partially paralyzed HSV-1-infected cotton rat. Female cotton rat infected at 6 weeks of age with 5 log_10_ PFU HSV-1 via lip abrasion was sacrificed on day 9 postinfection, when signs of paralysis of the right side of the body were detected. (A) Right side of the cerebellum of that animal. The two panels correspond to H&E- (left) and LFB- (right) stained consecutive sections of the same area. Thick arrows point to a broad demyelinated area in the cerebellar medulla. Arrowheads indicate perivascular cuffing. Asterisks are placed next to the areas of leptomeningitis. (B) Left side of the forebrain/midbrain of the same animal, stained with H&E (left) or LFB (right). Arrows point to smaller demyelinated lesions in thalamus (Th) and cerebral peduncle (Cp). Arrowheads indicate perivascular cuffing. 3V, third ventricle; pc, posterior commissure. Magnification, ×20.

HSV-1 infection of cotton rats infected via lip abrasion was associated with two main types of effects, namely, morbidity/mortality which was usually preceded by hunched posture, compromised balance, and/or splayed hind feet, and a milder manifestation in the form of defective blink and whisker touch reflex, some facial paralysis, and head tilt from which the majority of animals recovered (Tables 1 and 2). All animals with morbidity/mortality for which histologic samples were available had demyelinated lesions in the brain (Table 1). The lesions usually involved both the BST and cerebellum and occasionally the forebrain/midbrain. A particularly severe case of demyelination and inflammation was detected in one study in which an animal with a paralysis of the right side of its body was found (study 3 in Table 1). The change was noticed during a routine health check on day 9 postinfection. The right side of the body of that animal appeared to be paralyzed, and the animal was dragging its body in circles around the affected side. Aside from the lesions in the BST/pons, severe, locally extensive demyelination and necrosis were present in areas of cerebellar white matter tracts (Fig. 6). Milder lesions were present in the cerebral peduncle (crus) and thalamic nuclei. Perivascular inflammatory cell infiltrates, including numerous granulocytes, were widespread. Experiments are under way to correlate milder symptoms of HSV-1 infection (shown in Table 2) with the extent of brain damage. Preliminary studies indicate that while individual demyelinated lesions in the BST/pons may be asymptomatic, the presence of multiple lesions in the BST/pons and cerebellum may be associated with symptoms (Table 3).

**TABLE 3:** Brain histopathology in cotton rats with or without head tilt^*a*^

Sex^*b*^	Lesion results by brain area									Symptom(s)
	BST/pons			Cerebellum				Forebrain/midbrain		
	Right ST DemLesn	Non-ST DemLesn	Perivasc	DemLesn	Total no. of DemLesn	Perivasc	Leptom	Perivasc	DemLesn	
F	+	−	−	−		−	−	+	−	None
F	+	−	−	+	1	−	+	++	−	None
F	+	−	+	+	5	++	+	++	−	FA, RT−, RB−, HT
M	+	−	−	−		−	+	−	−	None
M	+	−	+	+	1	+	+	+	−	HT
M	+	2	+	+	10	+	+	+	−	FA, RT−, RB−, HT

a Female and male cotton rats, 5 to 6 weeks old, were infected with 5 log_10_ PFU HSV-1 in the lip via abrasion and monitored for symptoms as described in the footnote to Table 2. On day 10 postinfection, 6 of the animals who did or did not display head tilt were sacrificed and brains were collected for histopathology analysis. Demyelinated lesions (DemLesn), perivasculitis (Perivasc), and leptomeningitis (Leptom) were evaluated in BST/pons, cerebellum, and forebrain/midbrain and their presence is indicated by “+” for each brain area separately. “Right ST” denotes sensory trigeminal area of the right side of BST/pons that includes trigeminal root entry zone (TREZ), sensory root of the trigeminal nerve, and principal and spinal trigeminal nuclei; “non-ST” denotes all other areas of BST/pons. Total number of demyelinated lesions detected in different parts of the brain is shown. Notation of inflammatory changes corresponds to absent (−), moderate (+), or strong (++). Mild symptoms are abbreviated as described in the footnote to Table 2.

b F, female; M, male.

### Effect of vaccination against HSV-1 on brain infection in cotton rats.

**FIG 7:**
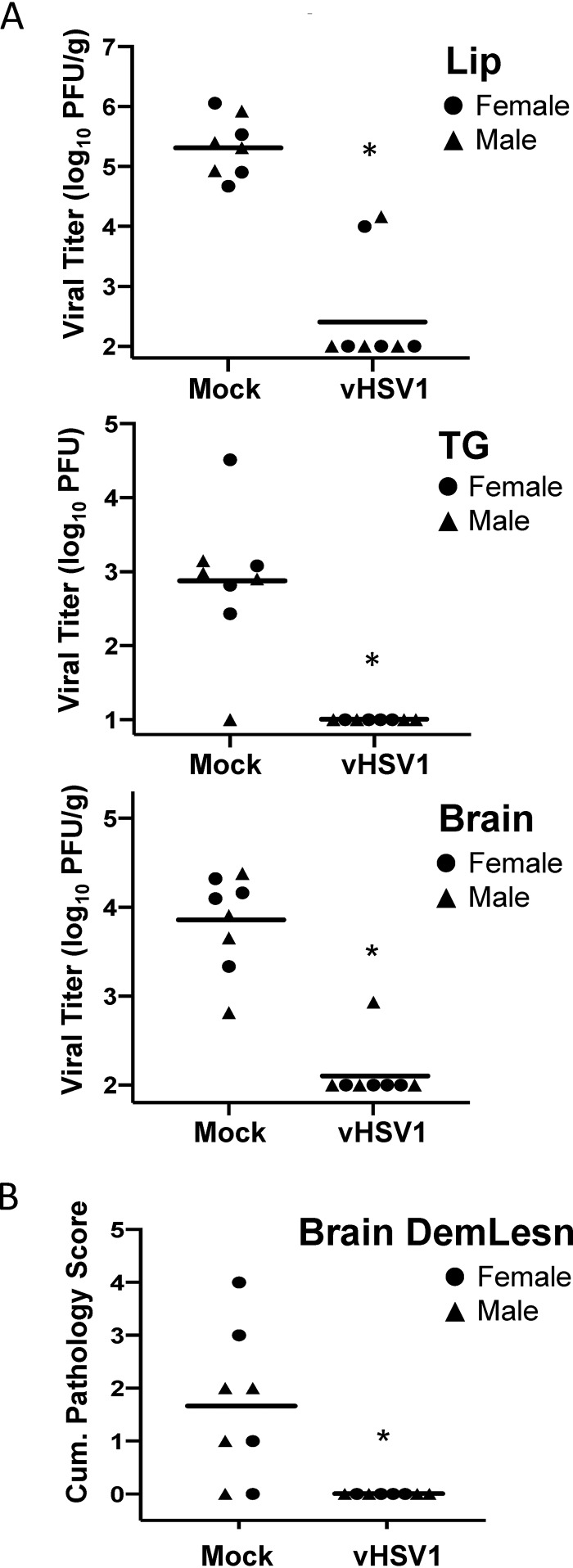
Prevention of HSV infection and brain pathology by vaccination against HSV-1. Young cotton rats (males and females, 4 to 5 weeks old) were vaccinated with a split HSV-1 vaccine (vHSV1) or mock vaccinated and infected at 10 weeks of age with 5 log_10_ PFU HSV-1 via lip abrasion. (A) Groups of HSV-1-infected animals (4 females and 4 males vaccinated with either vHSV1 or mock) were sacrificed on day 5 postinfection for analysis of viral load in the lip, TG, and brain. (B) Brain histopathology was evaluated for the presence of demyelinated brain lesions (DemLesn) on day 13 postinfection in 4 females and 4 males per group. Cumulative score for demyelinated lesions in BST/pons and cerebellum is shown. No lesions were detected in forebrain/midbrain. Individual female and male animals are represented as circles and triangles, respectively. Statistical significance of differences between mock- and vHSV1-vaccinated animals was assessed by one-way ANOVA followed by Tukey *post hoc* test. *, *P* < 0.05. Experiment was repeated once using female animals, with similar results.

To determine if vaccination against HSV-1 can reduce brain viral load and pathology in HSV-1-infected animals, a split HSV-1 vaccine (vHSV1) was prepared. Female and male cotton rats were immunized at the age of 4 to 5 weeks, boosted 3 weeks later, and infected with HSV-1 in the lip via abrasion at ∼10 weeks of age. Virus was detectable in the lip, brain, and TG of all mock-immunized, HSV-1-infected animals, with the exception of one male who did not show virus in TG (Fig. 7A). Demyelinated lesions were visible in the brain of all HSV-1-infected mock-immunized animals, with the exception of one male and one female (Fig. 7B). Immunization of cotton rats with the split HSV-1 vaccine significantly reduced viral replication in the lip, TG, and brain (Fig. 7A). HSV-1 replication was still detectable in the lip of two cotton rats (one male and one female) and in the brain of one male animal immunized with vHSV1 and challenged with HSV-1. HSV-1 vaccine significantly reduced brain pathology, with no demyelinated lesions visible in the brain of any animal (males or females) that received vHSV1 (Fig. 7B). No sex-specific effect of vaccination on viral load or vaccine efficacy against brain demyelination was noted. These results indicate that vaccination against HSV-1 can reduce virus presence in the CNS and associated brain pathology after acute HSV-1 infection.

## DISCUSSION

The work described here demonstrates that cotton rats infected with HSV-1 via a common route (infection in the lip) support active replication of virus at the site of inoculation and allow for efficient transfer of virus to the trigeminal ganglia with further progression of infection into the brain, with signs of encephalitis, meningitis, and multifocal CNS demyelination. Virus can be reactivated following application of stress stimulants, such as corticosteroids. Although conditions for HSV-1 reactivation in the cotton rat model still need to be further optimized, our results demonstrate that primary infection, reactivation, and HSV-1-induced CNS damage, including inflammation and multifocal demyelination, can be modeled in cotton rats, *S. hispidus*. Moreover, preliminary studies conducted using an HSV vaccine (split preparation) further suggest that brain pathology is a consequence of HSV-1 infection in the model and can be reduced by methods that target HSV-1 infection.

Similar to what has been reported for HSE (16, 17), there was some mortality, perivascular lymphocytic cuffing in pons and forebrain, thalamic and cerebellar involvement, and meningitis in HSV-1-infected cotton rats. Mortality and morbidity manifestations in some of the HSV-1-infected cotton rats (hunched posture and compromised balance) are similar to the symptoms of HSE in mice (50–52). The extent of meningitis detected in this work may be underrepresented. In addition to the fact that dura mater usually sticks to the calvarium during sampling in rodents, cotton rat brains in this study were immersed in formalin after extraction from the animal, rather than being collected after full-body formalin perfusion, potentially further compromising meningeal integrity. The whole body perfusion could not be used in this work, as it would inactivate virus and compromise the goal of measuring HSV-1 replication and correlating it with changes in the CNS.

One of the most important findings of our work has been the detection of multifocal CNS demyelination in cotton rats infected with HSV-1. Virus antigens were detected in association with demyelinated lesions, suggesting that demyelination, at least in part, is a direct effect of viral infection/presence in the brain tissue and that demyelination is a consequence of primary HSV-1 infection. Multifocal CNS demyelination following HSV-1 infection could not be achieved before in a rodent model that has an intact immune/inflammatory system and required the use of murine strains genetically prone to developmental defects, tumorigeneses, and autoimmune disorders. Regular cotton rats, *S. hispidus*, are not prone to these disorders and, instead, have proven to be a reliable translational model for multiple infectious diseases that affect humans (47–49). The fact that multifocal CNS demyelination can be induced in cotton rats upon inoculation with HSV-1 in the lip suggests that a lip HSV-1 infection in humans may lead to multifocal CNS demyelination. The process of remyelination rapidly followed demyelination in the brains of HSV-1-infected cotton rats, resulting in the formation of partially remyelinated plaques resembling shadow plaques where lesions have regained some of their LFB staining. These findings highlight a link between HSV-1 infection and CNS demyelination and provide a potential evidence for infectious etiology of demyelinating diseases like MS and ADEM. The time course of primary HSV-1-induced lesions and pattern of progression into CNS may be conserved among different species. Similar to what was seen in susceptible murine strains (46, 53), demyelinated lesions in cotton rats were detected in the BST 7 to 8 days after initial lip infection and progressed in the sequence BST > cerebellum > cerebral hemispheres. Demyelinated lesions in susceptible murine strains may resemble those seen in cotton rats, as they are also characterized by the loss of LFB-positive material and mononuclear cell infiltrate (45, 46). The size and number of lesions in cotton rats appear to be more similar to PL/J mice, where large and numerous lesions were detected, than to SJL/J mice, where lesions were small and few in number (46). To the best of our knowledge, no demyelinated lesions have been described in cotton rats before, so no comparison of HSV-1-induced changes to other types of demyelinated lesions in the model is possible.

Clinical symptoms of MS include defects in sensation, coordination, vision, and muscle weakness, with the exact nature of symptoms dependent on the location of demyelinating lesions within the nervous system. MS involvement of deep white matter tracts in the cerebrum of humans does not fit the more acute lesions described in this work. However, the human cases usually represent clinical examples of a more severe long-term disease process, and this work represents an acute disease limited by a month-long interval. It is also possible that the demyelination in the cotton rat model of HSV-1 infection represents a specific subset of MS cases. Detectable lesions in TREZ account for approximately 10% of lesions in MS patients (54). Trigeminal nerve disorders, including trigeminal neuralgia at the BST level and trigeminal paralysis, are common signs of MS. The mild neurologic defects detected in some HSV-1-infected cotton rats in the form of blink and touch response deficiency, facial paralysis, and transient head tilt could be representative of the cerebellum and/or BST dysfunction due to demyelination. Defective corneal blink reflex has been detected in patients with trigeminal neuralgia (55–57). Corneal blink reflex involves a loop between the trigeminal sensory nerves and the facial motor nerve, with signals relayed through the spinal trigeminal nucleus and the facial nucleus in the BST. Recent electrophysiological studies also reveal the communication between trigeminal and facial nerves at the level of the solitary nucleus of the facial nerve in medulla and lower pons (58). Demyelination in the corresponding areas of BST, thus, could affect blinking and other motor function in the face. Changes in posture and balance have also been seen in severely morbid cases of HSV-1 infection in cotton rats. While these are reported symptoms of HSE in mice (50–52), the loss of balance is also a frequent consequence of ADEM in humans, with lesions in cerebellum and BST reported (59, 60).

The majority of early changes identified in HSV-1-infected cotton rats were visible in the right TREZ and principal and spinal trigeminal nuclei. This location is consistent with the detection of virus in trigeminal ganglia prior to its appearance in the pons and reiterates that after lip scarification, virus enters the brain via the trigeminal nerve. The detection of additional, less frequent lesions and inflammation in the left cerebral peduncle and left thalamus suggests that virus may ascend the trigeminal lemniscus pathway past the second-order neurons. The detection of infectious HSV-1 in the forebrain/midbrain after the cerebellum and the BST/pons supports this hypothesis. Lesions were occasionally seen in the area corresponding to the solitary tract, suggesting that not only the facial nerve but also the glossopharyngeal nerve and the vagus nerve could be affected. Interestingly, oculomotor disorders (61), glossopharyngeal neuralgia (62, 63), and vagus-associated cognitive fatigue and autonomic abnormalities (64) have all been described in MS patients. The cross communication between different neuroanatomical structures in the BST can be very extensive. HSV-1 entering the BST along one of these pathways (e.g., trigeminal) may be able to affect multiple structures that happen to lie in physical proximity to the lesion created by infection. Overall, it may be important to consider that HSV-1-induced demyelinating lesions in the BST, cerebellum, and forebrain may have a broad damaging effect on a variety of neurological structures, leading to a multitude of symptoms and associated disorders.

Neurologic manifestations in the form of facial asymmetry, defective blink and whisker touch responses, and head tilt were first seen in cotton rats about a week after infection, suggesting that they may be associated with the increased virus presence in the brain, brain inflammation, and formation of demyelinated lesions. Almost all of the animals recovered from these symptoms. The timing of recovery from the symptoms (start on day 12 and completion by day 17) corresponds to the time when remyelination was seen in cotton rats’ brains. It is possible that in contrast to the severe morbidity/mortality detected in some HSV-1-infected animals, recovery from mild symptoms may correlate more directly with the process of remyelination in the cotton rat model. A preliminary comparison of animals that displayed those symptoms to asymptomatic animals showed that while demyelinated lesions in the BST/pons were detected in both symptomatic and asymptomatic animals, more extensive multifocal demyelination was seen in animals that developed symptoms. The correlation of behavioral abnormalities with brain pathology is a subject of ongoing work in our laboratory. It is possible that additional symptoms were missed and/or more animals had behavioral problems in the studies described here. In one case, an animal had a severe neurologic defect and partial paralysis of the entire right side of the body. The brain pathology in that animal was extensive, with the majority of the cerebellar medullar tissue destroyed by lesions and additional numerous demyelinated lesions and inflammation present in the contralateral cerebral peduncle, where the cortico-spinal tract is located, and the thalamus.

Earlier studies on lip HSV-1 infection in cotton rats (65) did not reveal any brain pathology, which may be associated with the choice of infectious strain, for example, HSV-1 strain F used before versus HSV-1 strain 17 used in the studies reported here or differences in the method of infection. In earlier studies, HSV-1 was inoculated in the center of the lower lip, while in this work it was inoculated in the vermilion border of the right side of the upper lip. The extensive lip lesions detected in the earlier work were not seen in this work, indicating that the method of lip infection and/or viral strain choice may influence manifestations of disease in the model. The actual method of virus delivery to the lip via abrasion used in this work could be important. Virus delivered via abrasion was more effective at replication in the cotton rat lip than the same dose of virus delivered subcutaneously (data not shown), even in the absence of visible lesions. These data may also suggest that some lip HSV-1 infections in humans could be even more asymptomatic than currently appreciated and explain the difficulty in drawing a parallel between HSV-1 and CNS sequelae in the absence of easily detectable signs of infection.

The sex and age of animals at infection may also need to be taken into consideration. There appeared to be a trend for more mortality/morbidity associated with HSV-1 infection in male versus female cotton rats in this work. Interestingly, although MS is diagnosed more frequently in women than in men, the disease can be associated with stronger manifestations and worse prognosis in males (66–68). Additional studies are needed to evaluate the effect of sex on HSV-1 disease pathogenesis in the cotton rat model, as most studies on disease pathogenesis reported here used females. The age of animals at the time of infection may also need to be considered. The majority of studies reported here were conducted using 5- to 6-week-old animals. A trend for increased disease manifestations was noted for cotton rats infected with HSV-1 at 5 to 6 weeks of age compared with animals infected at 10 to 12 weeks of age. While the age difference between these two groups does not appear to be dramatic, they may represent two different stages of brain development. Experimental evidence suggests that the brain of a 35- to 49-day-old rodent corresponds to the brain of a 12- to 18-year-old adolescent, while the brain of a >60-day-old rodent corresponds to the brain of a >20-year-old adult (69). While myelination is still ongoing in the adult brain, it is not as extensive as in the adolescent brain that undergoes synaptic pruning and needs to establish new circuitry. In recent years, pediatric MS, also called pediatric onset multiple sclerosis (POMS), has been gaining recognition. Pediatric MS is characterized by the first presentation of symptoms before the age of 18, with the highest incidence in 13- to 16-year-old adolescents (70, 71). Cerebellar and brain stem involvement are more common in pediatric onset MS than in its adult version. The incidence of pediatric MS is increasing worldwide (70), and although the disease has so far been linked with Epstein-Barr virus (EBV) (72), a possible connection between HSV-1 infection and pediatric MS may need to be explored in more depth.

## MATERIALS AND METHODS

### Cells and virus.

HSV-1 (strain 17) (kindly provided by Priscilla Shaffer of Harvard Medical) was grown on Vero cells and stored at –80°C at a concentration of ∼10^8^ PFU/ml. Virus was diluted in phosphate-buffered saline (PBS) (pH 7.4) to an appropriate concentration within an hour of infection and maintained on ice.

### Vaccine.

HSV-1 split vaccine was prepared using Vero cells infected with HSV-1. Cells were propagated in Eagle’s minimum essential medium (EMEM) supplemented with 10% fetal bovine serum (FBS), 1% l-glutamine, gentamicin, and fungizone and infected with HSV-1 at a multiplicity of infection (MOI) of 0.01. After 18 h of infection, cells were washed and lysed with 1% IGEPAL in Tris-NaCl-EDTA buffer. The cell solution was subjected to one cycle of freeze-thawing and clarified by centrifugation at 4,000 × *g* at 4°C. Supernatants were filtered, recentrifuged, and dialyzed against PBS (pH 7.4) at 4°C. After an additional centrifugation, vaccine was aliquoted and stored at –80°C until use. For use in animals, vaccine was adjuvanted with 2% squalene oil-in-water emulsion with monophosphoryl lipid A and synthetic trehalose dicorynomycolate (Sigma adjuvant system). Mock vaccine was prepared following the same protocol but using Vero cells that were not infected with HSV-1.

### Animals.

Inbred Sigmodon hispidus cotton rats were obtained from a colony maintained at Sigmovir Biosystems, Inc. Four- to 10-week-old animals were used for the studies, with specific age and sex indicated in the figure legends and tables. Animals were housed in large polycarbonate cages and were fed a standard diet of rodent chow and water. The colony was monitored for antibodies to adventitious respiratory viruses and other common rodent pathogens, and no such antibodies were found. All studies were conducted under applicable laws and guidelines and after approval from the Sigmovir Biosystems, Inc., Institutional Animal Care and Use Committee (IACUC).

### Animal studies.

Cotton rats were anesthetized with ketamine-xylazine by injecting intramuscularly (i.m.) at 100 μl/100 g of body weight a mixture of 5 parts 100 mg/ml ketamine and 1 part 33 mg/ml xylazine. Animals were infected with HSV-1 in the lip by placing a 20-μl drop of virus on the vermilion border of the upper right lip, followed by repeat abrasion of the area using a sharp needle. At various times after infection, animals were sacrificed by CO_2_ asphyxiation for sample collection. Control animals were inoculated with 20 μl of PBS [pH 7.4] in the lip via abrasion. The right half of the upper lip, the right trigeminal ganglion, and the brain were extracted and homogenized in 500 μl (lip, TG) or 5 ml (brain) of EMEM with sucrose stabilizing solution for viral titration (homogenization buffer). For studies on viral detection in different part of the brain, cotton rats were infected with 20 μl containing 5 log_10_ PFU HSV-1 via abrasions and sacrificed on days 3 and 5 postinfection. Brains were extracted and dissected into the following three portions: brain stem/pons, cerebellum, and forebrain/midbrain. Each section was homogenized in 1.67 ml of homogenization buffer. Brains for histopathology analysis were extracted and immersed in 10% buffered formalin. For studies on reactivation, animals were inoculated intramuscularly with 40 mg/kg triamcinolone (Kenalog-40) 1 month after the original HSV-1 infection and sacrificed at various times after treatment. Observational studies on mild symptoms were conducted using male and female animals infected with 5 log_10_ PFU HSV-1 via lip abrasion. Symptoms included head tilt, loss of blink reflex and whisker touch response, and facial asymmetry. Blink reflex was evaluated by blowing air from a syringe into the right and left eye and recording successful or unsuccessful blinking on each side. Whisker touch response was evaluated by touching whiskers on the right and left side with a cotton tip applicator and monitoring twitching. Blink reflex and whisker touch response were evaluated three times on each side; head tilt and facial asymmetry were examined visually. Animals were observed for 19 days after infection and onset of and recovery from symptoms were recorded. Animals for the study on HSV-1 vaccination were immunized with the adjuvanted split vaccine intramuscularly (100 μl per animal) twice in a period of 3 weeks, infected with 5 log_10_ PFU HSV-1 via lip abrasion 2 weeks after the second immunization, and sacrificed at various times after infection for analysis of viral replication and histopathology. Animal studies were performed and repeated as described in the figure legends.

### Virus titrations.

Lip, brain, and TG homogenates were assayed for the presence of infectious HSV-1 by titering homogenates on Vero cells by plaque assay. In brief, homogenates were clarified by centrifugation and diluted in EMEM. Confluent Vero monolayers were infected in duplicates with diluted homogenates in 24-well plates. After a 1-hour incubation at 37°C in a 5% CO_2_ incubator, cells were overlaid with 0.75% methylcellulose medium. After 2 days of incubation, the overlay was removed and the cells were fixed with 0.1% crystal violet stain for 1 hour, rinsed, and air dried. Plaques were counted, and virus titer was expressed as PFU per gram of tissue (lip and brain) or total PFU detected (TG). The limit of detection of the plaque assay was 2 log_10_ PFU/g for lip and brain and 1 log_10_ PFU for TG. The limit of detection for the plaque assay on brain pieces homogenized individually was 0.68 log_10_ PFU/g for forebrain/midbrain and 1.48 log_10_ PFU/g for brain stem and cerebellum.

### Brain histopathology.

Brain was extracted from animals immediately after euthanasia and submerged in 10% buffered formalin. Brains were cut into 4 coronal segments, embedded in paraffin, and sectioned. Slides were stained with hematoxylin and eosin (H&E) or Luxol fast blue (LFB). The following four coronal views of the brain were available for analysis: section 1, forebrain at the level of the frontal pole of the cerebrum or the rostral end of the corpus callosum; section 2, forebrain at the level of the anterior commissure; section 3, forebrain at the level of the caudal end of the thalamus and midbrain; and section 4, hindbrain at the level of the facial nucleus or the inferior olive. Sections 1 and 2 did not reveal pronounced changes associated with HSV-1 infection; therefore, the focus was made on sections 3 and 4, which contained BST/pons, cerebellum, or forebrain/midbrain. The presence of demyelinated lesions in the BST/pons, cerebellum, or forebrain/midbrain was analyzed and assigned a value of 0 (no lesion), 1 (single lesion), or 2 (multiple lesions). Perivascular cuffing in the BST/pons, cerebellum, or forebrain/midbrain and inflammation in leptomeninges in the cerebellum were assigned values 0 (absent), 1 (moderate), 2 (strong), or 3 (very strong). Cumulative pathology score for demyelinated lesions was calculated from demyelinated lesion scores for BST/pons, cerebellum, and forebrain/midbrain. Cumulative pathology score for inflammation was calculated from scores of perivascular cuffing in the BST/pons, cerebellum, and forebrain/midbrain and inflammation in leptomeninges in the cerebellum. In the absence of a detailed atlas of cotton rat brain, the location of cuts and corresponding structures was deduced based on the atlas of a Wistar rat brain (73).

### Brain immunohistochemistry.

Brain tissue was deparaffinized and rehydrated. Following heat-mediated antigen retrieval, tissue was permeabilized with 0.5% Triton in Tris-buffered saline (TBS) and blocked with 5% normal cotton rat sera in 1% BSA in TBS. Anti-HSV-1 rabbit polyclonal antibody (Abcam ab9533) in TBS with 1% BSA was added. After washing with TBS with 0.025% Triton, slides were treated with 0.01% H_2_O_2_ in TBS and incubated with goat anti-rabbit IgG antibody conjugated with horseradish peroxidase (HRP) (Abcam ab6721) in TBS with 1% BSA. Color was developed using DAB+ solution (Agilent Dako) followed by hematoxylin (Vector Laboratories). Slides were dehydrated and mounted in DPX mountant (Sigma-Aldrich).

### Statistical analyses.

Comparisons were performed by Student’s *t* test or analysis of variance (ANOVA), followed by Tukey *post hoc* test, as indicated.
